# Enhancing Quality of Life of Proximal Gastrectomy: A Comprehensive Review of Anastomotic Innovations and Challenges

**DOI:** 10.1002/cam4.71554

**Published:** 2026-02-04

**Authors:** Linchuan Li, Dexu Zhang, Siyi Song, Shuohui Dong, Qian Xu, Guangyong Zhang, Jiankang Zhu

**Affiliations:** ^1^ Department of General Surgery The First Affiliated Hospital of Shandong First Medical University & Shandong Provincial Qianfoshan Hospital Jinan Shandong China; ^2^ Department of General Surgery Shandong Provincial Qianfoshan Hospital, the First Affiliated Hospital of Shandong First Medical University, Laboratory of Metabolism and Gastrointestinal Tumor Jinan Shandong China; ^3^ Shandong Provincial Qianfoshan Hospital, Shandong University Jinan Shandong China; ^4^ Department of Anesthesiology The First Affiliated Hospital of Shandong First Medical University & Shandong Provincial Qianfoshan Hospital, Shandong Institute of Anesthesia and Respiratory Critical Medicine Jinan Shandong China; ^5^ Department of General Surgery Qilu Hospital of Shandong University Jinan Shandong China

**Keywords:** anastomotic complications, gastric cancer, proximal gastrectomy, reconstruction, reflux esophagitis, stenosis

## Abstract

**Background:**

In recent years, the incidence of early upper gastric cancer has increased, leading to a wider adoption of proximal gastrectomy (PG) as a possible treatment option. PG is preferred over total gastrectomy (TG) due to its superior postoperative nutritional status and improved surgical safety. However, PG is associated with a considerable risk of anastomosis‐related complications, such as gastroesophageal reflux and anastomotic stenosis, which limit its widespread application.

**Methods:**

We conducted a narrative review of comparative studies, prospective studies and retrospective study, that described reconstructive techniques after PG. End‐points of interest were incidence of reflux esophagitis, anastomotic stenosis, time of follow‐up, nutritional parameters.

**Results:**

Esophago‐gastrostomy, the simplest reconstruction method, was associated with highest reflux rates of 18%–55% and stenosis rates of 1.1%–27.8%. Anti‐reflux modifications such as side‐overlap, double‐flap technique (DFT) and gastric tube lowered reflux rate distinctly, especially DFT, lowered reflux rates to 2%–16.7%. But DFT carried a 4%–26.3% stenosis risk and longer operative time. Jejunal interposition (JI) gave 2.1%–100% reflux rates and 0%–31.8% stenosis, yet required three anastomoses and limited endoscopic surveillance. Double‐tract reconstruction (DTR) achieved the promising anti‐reflux outcome, with preserved duodenal passage. However, it may increase surgery costs and prolong surgical time.

**Conclusion:**

Our findings provide a solid foundation for reconstruction methods selection that enhance postoperative quality of life and highlight future directions for improving PG outcomes. DTR and DFT, currently offer the best balance between reflux control and anastomotic stenosis after PG. Prospective clinical research and innovation are expected to further improve these techniques and may discover an optimal universal approach to address long‐term challenges.

## 1 Introduction

Gastric cancer is the fifth most common cancer worldwide, with over 1 million new cases annually [1]. Deaths from gastric cancer have exceeded 760,000 each year, ranking as the fourth leading cause of the mortality rate of malignant tumors. Improvement in endoscopy has led to more early diagnoses of gastric cancer. Even though the overall incidence of gastric cancer is declining, the incidence of upper gastric adenocarcinoma or early esophagogastric junction adenocarcinoma has risen significantly in recent decades [2, 3, 4].

For most upper gastric cancer and esophagogastric junction cancer, except for a few early‐stage gastric cancer cases that are feasible for endoscopic resection, the majority of cases need surgical treatment. Traditionally, total gastrectomy (TG) is generally considered to be the standard treatment for cancer of the upper stomach, which is beneficial for complete lymph node dissection and avoidance of gastroesophageal reflux [5]. However, TG may represent overtreatment for early‐stage upper gastric cancer due to the promotion of gastroscopy application. With permanent loss of storage as well as secretory function of the stomach after TG, it usually causes postoperative malnutrition, which seriously affects patients' quality of life [6].

In contrast, proximal gastrectomy (PG) is a functional‐preserving alternative therapy, which only removes the upper part of stomach, while sparing the distal part. For appropriate cases at the early stage, PG could achieve the same oncological outcome as TG, and could also maintain the better postoperative nutritional status [7]. Therefore, PG has been feasible and safe to perform, to minimize surgical stress and preserve gastric function [8]. According to the Japanese gastric cancer treatment guidelines version 6, PG is recommended for treatment of cT1N0 adenocarcinoma located in the upper third of the stomach, with preservation of over half of the distal stomach [9]. Several studies have reported favorable nutritional outcomes with PG [10, 11]. PG preserves the grinding and digestive function of stomach, which prolongs gastric emptying and reduces the occurrence of postoperative dumping syndrome, with promising oncologic safety of sparing the distal stomach in early‐stage patients [12]. These findings have further aroused intensive interest in PG.

Although PG is quite attractive, there are still many complex and reconstructive challenges. Due to the loss of the anti‐reflux effect of the gastroesophageal junction and direct exposure of gastric acid on the anastomotic region, anastomotic‐related complications, such as gastroesophageal reflux and anastomotic stenosis, are major limitations of the widespread adoption of PG. To ameliorate this problem, variable reconstruction techniques, influenced by surgeon preference and factors, such as remnant stomach size, are employed. These include modifications to the esophagogastrostomy, jejunal interposition, and double‐tract reconstruction, among others. Although many new surgical procedures have been introduced, none has been broadly accepted, and controversy exists regarding the optimal digestive tract reconstruction after PG.

In light of this, we reviewed the anatomical basis and mechanism of action of the gastroesophageal junction and current reconstructive methods following PG. We performed a comprehensive literature search to collect all studies discussing reconstruction techniques after PG. The primary database used was PubMed. Key search terms included combinations of “gastric cancer,” “proximal gastrectomy,” “reconstruction.” We also conducted a manual screening of the references in the relevant literature to supplement our search content.

To ensure that there is sufficient data for meaningful analysis, we included studies with a sample size of more than 10 cases and which underwent specific reconstructive surgeries, with reported incidence of anastomotic stricture, reflux esophagitis, and other relevant information into tables, as smaller series studies or case reports provide less information for drawing universally applicable conclusions. Given the heterogeneity of outcome indicators, a quantitative meta‐analysis could not be conducted. Therefore, we summarize the findings in a narrative manner and present the complication rates of different techniques in a comparison table. Abstracts, case reports, and non‐English articles were not included in this review.

The following text will first briefly review the relevant anatomy of the esophagogastric junction, then describe the common postoperative reconstruction methods and their new modifications, and subsequently critically discuss the significance of the findings and conclusions.

## 2 Review of the Anatomy and Function of Gastroesophageal Junction

**FIGURE 1:**
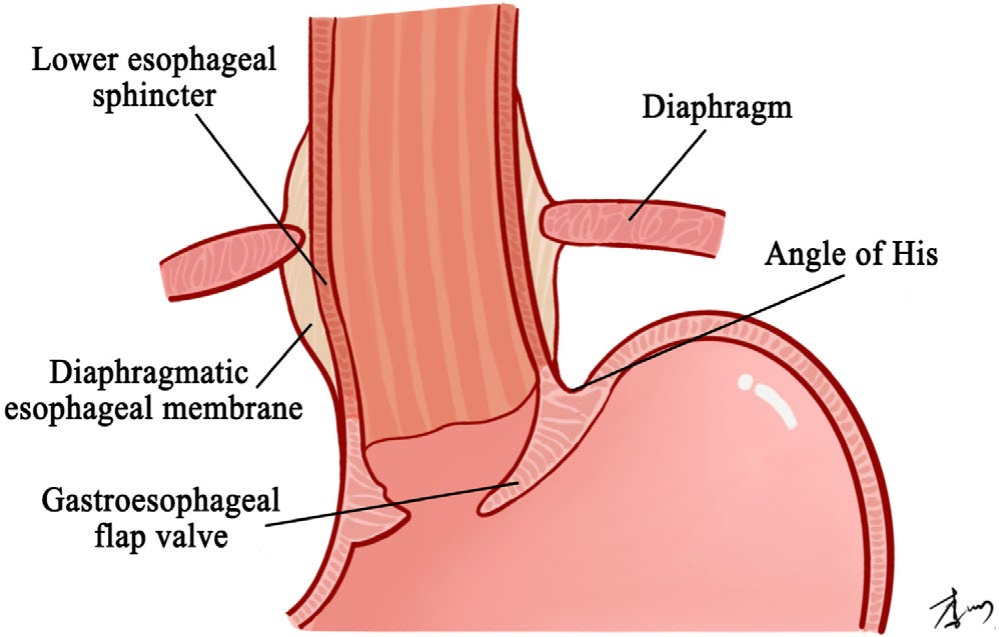
Anatomical structures of the gastroesophageal junction.

Gastroesophageal junction is the anatomical interface between the distal esophagus and proximal stomach (Figure 1), comprising essential anatomical components that are crucial for maintaining normal digestive processes and preventing acid reflux [13].

### 2.1 Lower Esophageal Sphincter

The lower esophageal sphincter (LES) is a muscular structure consisting of gastric sling fibers and semicircular clap‐like fibers, which creates an internal high‐pressure zone identifiable through manometry. Approximately 2–4 cm in length, the LES encircles the gastroesophageal junction akin to a band. The three‐dimensional pressure measurement conducted in a study indicates that the highest pressure is observed in the left rear region, aligning with the orientation of the two muscles comprising the LES [14]. In individuals without esophageal disorders, the resting pressure of the LES typically ranges from 10 to 30 mmHg, exceeding the internal gastric pressure by 5–10mmHg [15]. During contraction, the LES can generate high pressure, which tightly closed gastroesophageal junction [16, 17]. The LES relaxes upon food entry into the stomach via the esophagus, and contracts during normal conditions to prevent the reflux of gastric contents, acting as a physiological sphincter to maintain barrier function.

### 2.2 Diaphragm

The diaphragm aligns with the longitudinal axis of the spine, originating from the first to the third lumbar vertebrae and the intervertebral fibrocartilages. Typically, the right crus of the diaphragm forms the esophageal hiatus, with approximately 20% of anatomical variations involving the left crus [18]. Consequently, the distal esophagus typically traverses the diaphragmatic hiatus, while the diaphragm could function as an external sphincter by exerting pressure on the gastroesophageal junction [19].

### 2.3 Diaphragmatic Esophageal Membrane

The diaphragmatic esophageal membrane is composed of two lobes that originate from the diaphragm, with one lobe arising from the diaphragmatic surface of the thoracic cavity and the other lobe originating from the abdominal cavity. The upper lobe extends 2–4 cm above the diaphragm along the distal esophagus, with fibers inserting into the submucosa of the esophagus. The other lobe descends through the cardia and integrates with the gastric serosa and muscles. The two lobes penetrate the connective tissue between the longitudinal and circular muscles at the esophagogastric junction to maintain proper alignment of the diaphragm and LES during contraction [20].

### 2.4 Angle of His

The angle of His is the angle formed by the abdominal esophagus and gastric fundus, typically presenting as an acute angle. Contraction of LES results in a reduction of the His angle, leading to increased pressure of the gastroesophageal junction [21].

### 2.5 Gastroesophageal Flap Valve

The gastroesophageal flap valve (GEFV), characterized by the oblique intracavitary angle between esophagus and stomach formed by the angle of His, is a dynamic anatomical structure that plays a significant role in the pathophysiology of gastroesophageal reflux disease. Hill conducted studies utilizing cadaver and live subjects to confirm the presence of GEFV and introduced Hill's classification, which categorizes GEFV into four distinct levels based on endoscopic observations of its various forms [22]. The GEFV serves as a crucial component of the anti‐reflux barrier at the gastroesophageal junction, contributing to the maintenance of the internal and external sphincters at this anatomic site [23].

Based on the above anatomical structures, an important anti‐reflux mechanical barrier is formed in the gastroesophageal junction to prevent the reflux of stomach contents. The critical change caused by PG is loss of cardia, which plays an extremely important role in anti‐reflux mechanisms. In addition, a small amount of food after the removal of the gastric fundus and the adjacent part of the stomach could cause a significant increase in gastric pressure, and gastric emptying is delayed to some extent due to the preserved pylorus, thus leading to the occurrence of reflux esophagitis [24]. A deep and full understanding of these structures and their functions are of vital significance for the reconstruction after PG.

## 3 Common Reconstruction Methods after Proximal Gastrectomy

According to the 6th edition of the Japanese Gastric Cancer Diagnosis and Treatment Guidelines issued by the Japanese Gastric Cancer Association in 2021, three reconstruction methods after PG are recommended, which are esophagogastrostomy, jejunal interposition, and double‐tract reconstruction [9].

### 3.1 Esophagogastrostomy

**FIGURE 2:**
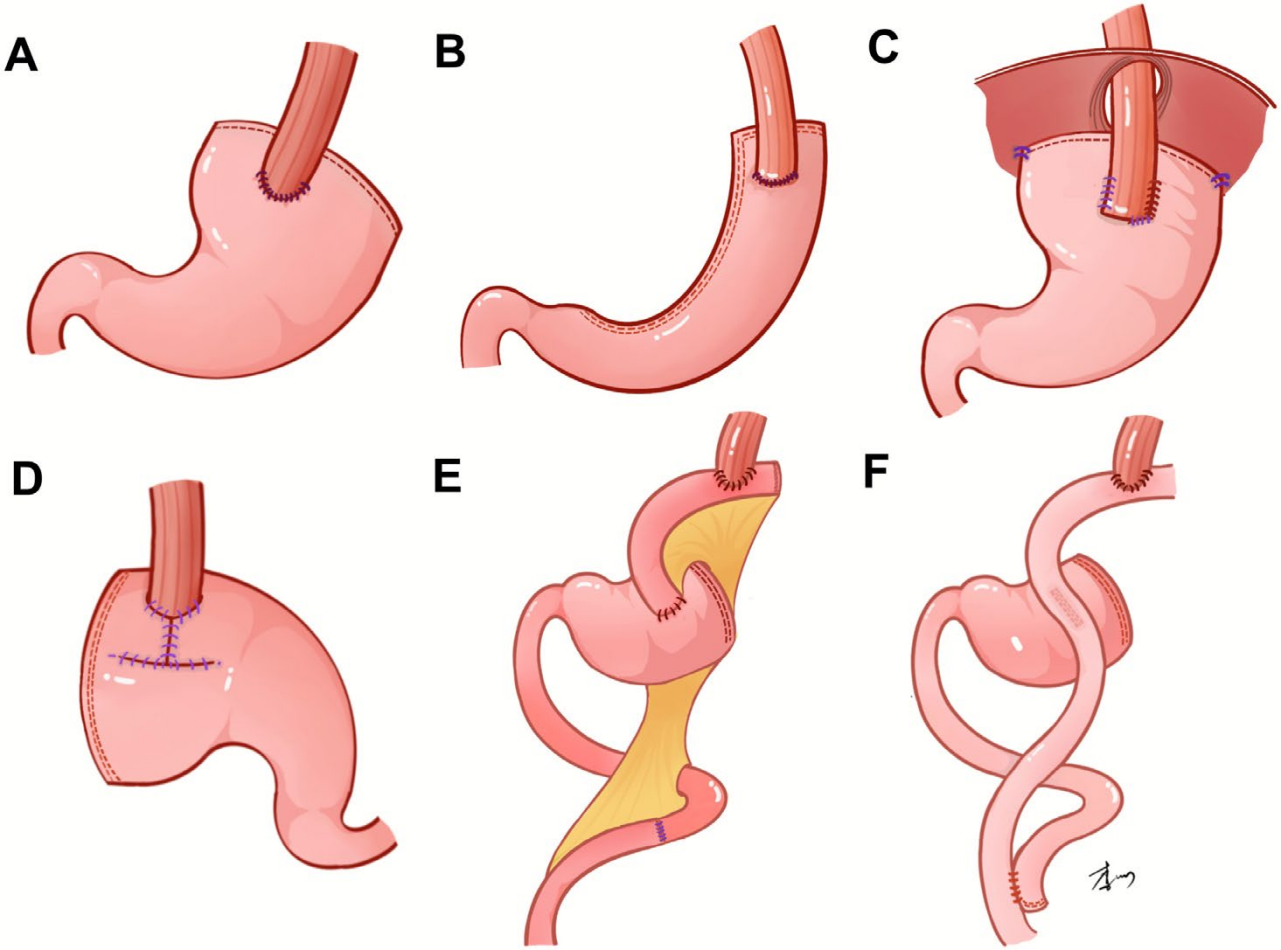
Different reconstruction methods of proximal gastrectomy. (A) Esophagogastrostomy. (B) Gastric tube reconstruction. (C) Side overlap. (D) Double‐flap technique. (E) Jejunal interposition. (F) Double tract reconstruction.

Esophagogastrostomy (EG) has been the most common reconstruction method used after PG (Figure 2A). Based on traditional EG, many surgeons also developed a variety of novel EGs with anti‐reflux function, which we describe in detail later.

#### 3.1.1 Traditional Esophagogastrostomy

EG is the simplest reconstruction method, which can be divided into two types depending on whether the gastroesophageal anastomosis is in the anterior or posterior wall of the stomach. EG is advantageous in simplicity and safety, in line with the physiological structure of the digestive tract, and lower overall cost.

Due to the direct acid exposure of the anastomotic site and disruption of the anti‐reflux structure of the esophagogastric region, neither anterior nor posterior anastomosis obtains desired anti‐reflux effect (Table 1). Thus, severe reflux esophagitis and anastomotic stenosis have been reported as the main complications of EG, with the incidence of both adverse events as high as 30% or higher [35, 37, 38, 39]. Therefore, EG has been used mainly in relatively unadvanced medical centers or for elderly or ill patients who could not tolerate more time‐consuming operations.

**TABLE 1:** The incidence of anastomotic disorders among esophagogastrostomy.

Authors	Country	Published year	Anastomotic method	Number of patients	Incidence of stenosis	Reflux symptom	Number of endoscopy	Total reflux esophagitis	Grade A	Grade B	Grade C	Grade D	Time of follow up (months)
Wang et al. [25]	China	2024	Esophagogastrostomy	96	0	43.8% (42/96)	96	43.8% (42/96)	N/A	N/A	N/A	N/A	N/A
Wei et al. [26]	China	2023	Esophagogastrostomy	93	1.1% (1/93)	N/A	60	55% (33/60)	N/A	N/A	N/A	N/A	12
Chen et al. [27]	China	2023	Esophagogastrostomy	51	2% (1/51)	45.1% (23/51)	51	37.3% (19/51)	21.6% (11/51)	9.8% (5/51)	5.9% (3/51)	0	12
Gong et al. [28]	China	2023	Esophagogastrostomy	36	27.8% (10/36)	30.6%^a^ (11/36)	36	33.3% (12/36)	N/A	N/A	N/A	N/A	12
Eom et al. [29]	Korea	2021	Esophagogastrostomy	45	24.4% (11/45)	N/A	45	17.8% (8/45)	6.7% (3/45)	11.1% (5/45)		N/A	12
Yamashita et al. [30]	Japan	2017	Esophagogastrostomy	16	18.8% (3/16)	N/A	16	31.3% (5/16)	N/A	N/A	N/A	N/A	12
Aburatani et al. [31]	Japan	2017	Esophagogastrostomy	22	27.3% (6/22)	54.5% (12/22)	22	31.8% (7/22)	9.1% (2/22)	9.1% (2/22)	13.6% (3/22)	0	12
Chen et al. [32]	China	2012	Esophagogastrostomy	41	22% (9/41)	34.1% (14/41)	41	22% (9/41)	14.6% (6/41)	4.9% (2/41)	2.4% (1/41)	0	12
Sugita et al. [33]	Japan	2021	Esophagogastrostomy with fundoplication	60	11.7% (7/60)	23.3% (14/60)	60	26.7% (16/60)	11.7% (7/60)	3.3% (2/60)	11.7% (7/60)	3.3% (2/60)	72
Tominaga et al. [34]	Japan	2021	Esophagogastrostomy with fundoplication	39	2.6% (1/39)	20.5% (8/39)	39	20.5% (8/39)	N/A	20.5% (8/39)			12
Hosoda et al. [35]	Japan	2019	Esophagogastrostomy with fundoplication	51	27.5% (14/51)	51% (26/51)	51	13.7% (7/51)	N/A	13.7% (7/51)			12
Nakamura et al. [36]	Japan	2014	Esophagogastrostomy with fundoplication	64	21.8% (12/55)	N/A	55	27.3% (15/55)	5.5% (3/55)	14.5% (8/55)	7.3% (4/55)	0	12

^a^ Reflux symptom classified according to the Visick grade. Values are Visick III or more.

#### 3.1.2 Gastric Tube Reconstruction

Gastric tube reconstruction (GT) was first reported by Shiraishi in 1998 [40]. In this operation, the remnant stomach is made into a tube with use of a linear stapler device followed by anastomosis of the gastric tube to the esophagus (Figure 2B). The anastomosis is made either end‐to‐side with use of a circular stapler or side‐to‐side with use of a linear stapler [41]. Formation of the narrow gastric conduit reduces the volume of stomach and decreases the secretion of gastric acid. The remnant end of esophagus is laid above the head‐shaped end of the gastric tube, forming a pseudo‐fundus, which reshapes the His angle [42]. These features improve nutrition status, as reflected by patients' serum total protein, and albumin values postoperatively [43]. Also, the narrow gastric tube leads to a rapid increase in gastric pressure when stomach is filled, which facilitates gastric emptying and restores gastrointestinal motility [44].

Based on these advantages, GT may favor satisfactory results in anti‐reflux (Table 2). Ronellenfitsch's group reported, although, that over 30% of patients with GT have reflux symptoms, while symptoms are mostly mild and without significant esophagitis [52]. Aihara and colleagues have reported a 14% incidence of gastroesophageal reflux after GT and a relatively high rate of anastomotic stricture (35%) [39]. A study that focused on the long‐term outcomes of GT revealed that 15.4% of patients had reflux esophagitis as diagnosed with endoscopy 1 month after surgery, but only 9.5% still suffered from reflux 1 year postoperatively. The anastomotic stenosis occurred in 13.0% after GT, which needs further balloon dilatation [50]. Several other studies have had similar results [32, 53].

**TABLE 2:** The incidence of anastomotic disorders among gastric tube reconstruction.

Authors	Country	Published year	Anastomotic method	Number of patients	Incidence of stenosis	Reflux symptom	Number of endoscopy	Total reflux esophagitis	Grade A	Grade B	Grade C	Grade D	Time of follow up (months)
Li et al. [45]	China	2024	Gastric tube	18	0	22.2% (4/18)	8	37.5% (3/8)	12.5% (1/8)	0	25% (2/8)	0	12
Wei et al. [26]	China	2023	Gastric tube	98	2% (2/98)	N/A	65	29.2% (19/65)	N/A	N/A	N/A	N/A	12
Chen et al. [27]	China	2023	Gastric tube	77	5.2% (4/77)	39% (30/77)	77	23.4% (18/77)	7.8% (6/77)	9.1% (7/77)	6.5% (5/77)	0	12
Xu et al. [46]	China	2023	Gastric tube	69	9.1% (5/55)	20%^a^ (11/55)	55	18.2% (10/55)	N/A	N/A	N/A	N/A	12
Hosogi et al. [47]	Japan	2022	Gastric tube	24	16.7% (4/24)	95.8% (23/24)	21	57.1% (12/21)	14.3% (3/21)	23.8% (5/21)	0	19% (4/21)	12
Li et al. [48]	China	2021	Gastric tube	151	0	13.2%^a^ (20/151)	151	31.8% (48/151)	19.9% (30/151)	8.6% (13/151)	3.3% (5/151)	0	3
Toyomasu et al. [49]	Japan	2021	Gastric tube	102	9.8% (10/102)	N/A	102	16.7% (17/102)	16.7% (17/102)				12
Toyomasu et al. [50]	Japan	2017	Gastric tube	84	13.1% (11/84)	N/A	84	9.5% (8/84)	6% (5/84)	3.6% (3/84)	0	0	12
Ueda et al. [41]	Japan	2016	Gastric tube	13	0	0	13	23.1% (3/13)	0	7.7% (1/13)	15.4% (2/13)	0	12
Yasuda et al. [51]	Japan	2015	Gastric tube	25	21.7 (5/23)	4.3% (1/23)	22	36.4% (8/22)	22.7% (5/22)	4.5% (1/22)	9.1% (2/22)		12
Ronellenfitsch et al. [52]	Canada	2015	Gastric tube	42	0	24.2% (8/33)	24	29.2% (7/24)	N/A	N/A	N/A	N/A	≥ 6
Chen et al. [32]	China	2012	Gastric tube	35	11.4% (4/35)	14.3% (5/35)	35	5.7% (2/35)	5.7% (2/35)	0	0	0	12
Adachi et al. [53]	Japan	1999	Gastric tube	14	7.1% (1/14)	7.1% (1/14)	14	7.1% (1/14)	N/A	N/A	N/A	N/A	N/A
Xu et al. [54]	China	2022	Modified gastric tube	74	1.4% (1/74)	5.4% (4/74)	74	5.4% (4/74)	1.4% (1/74)	4.1% (3/74)	0	0	6
Kanaji et al. [55]	Japan	2022	Modified gastric tube	30	0	96.7% (29/30)	30	46.7% (14/30)	10% (3/30)	23.3% (7/30)	13.3% (4/30)	0	12
Hosogi et al. [56]	Japan	2014	Modified gastric tube	15	20% (3/15)	0	13	30.8% (4/13)	N/A	N/A	N/A	N/A	12

^a^ Reflux symptom is classified according to the Visick grade. Values are Visick III or more.

A group of Chinese scholars has reported that patients who underwent GT also have digestive discomfort and other symptoms in addition to reflux [57]. Therefore, many modifications of GT have been developed in attempts to reduce the incidence of anastomotic‐related complications. In one operation, a Y‐shaped anastomosis is formed with the remnant esophagus and gastric tube connected at an angle of 60°, thus forming a pseudo‐fornix and His angle. The incidence of postoperative reflux symptoms was 13%, with no reported stenosis [55]. An alternative approach to GT positions the tip of the tube in the mediastinum to create a pseudo‐fornix and establish a stable angle of His, with satisfactory reflux consequence (4%) and lower anastomotic stenosis status (21.7%) [51]. Besides, Chinese surgeons have developed a novel mode of GT, in which the gastric fundus and His angle were reconstructed vertically at the distal end of the tubular stomach 2 cm away from the lesser curvature of the stomach, forming a giraffe‐like remnant stomach. This procedure had a 4.1% incidence of reflux esophagitis with Los Angeles (LA) grade B, with only one case of stenosis among 74 patients [54].

However, due to the requirement of dividing the gastric body into a conduit shape through linear staples, the expense of operation is higher than with other construction methods. Besides, the conventional gastric tube is about 20 cm long, making it mainly suitable for patients with high gastric margin.

#### 3.1.3 Side Overlap

Side overlap, first described by Yamashita in 2017, has also been called side overlap with fundoplication (SOFY) [30]. In this operation, the left side of the esophagus is sutured to the gastric stump by using a linear stapler, while the artificial fundus presses the dorsal esophageal wall as to maintain a valve function (Figure 2C). Side overlap is favored for its relative simplicity in laparoscopic operation. In addition, this method requires at least 5 cm overlap of the gastric stump with the esophagus, which may prevent the occurrence of anastomotic stenosis. This procedure also preserves the physiological and anatomical structures of the stomach, which may help maintain good postoperative nutritional status.

As reported, the incidence of esophagitis with side overlap was significantly lower than with EG (10% for side overlap group and 31.25% for EG group), and no anastomotic stenosis was observed in the side overlap group [30]. In some patients, anastomotic displacement was found during follow‐up, resulting in insufficient overlap between the lower esophagus and the anterior wall of the residual stomach and poor esophageal closure under increased artificial fundus pressure (Table 3). Therefore, Yamashita's group further modified this method, which made the anastomosis of the right side of the esophagus to the remnant stomach, so that the preserved esophageal wall stuck flat to the gastric wall as a valve. The modified method maintained a remarkable anti‐reflux effect with an incidence of esophagitis of 10.7% for LA grade B or higher, and the esophagitis only occurred near the anastomotic site, and few reflux symptoms were observed [60]. Chinese surgeon Wu made similar conclusions as above, in which only 2 of 20 patients who underwent modified SOFY developed mild reflux esophagitis, and only 1 patient developed anastomotic stenosis in the 1‐year follow‐up [61]. Hosogi's group devised a novel reconstruction with a combination of a gastric tube and SOFY. This approach is favorable for its simplicity and wide surgical field in the narrow mediastinum, which may have a potential anti‐reflux effect, although it needs further verification [47].

**TABLE 3:** The incidence of anastomotic disorders among side overlap.

Authors	Country	Published year	Anastomotic method	Number of patients	Incidence of stenosis	Reflux symptom	Number of endoscopy	Total reflux esophagitis	Grade A	Grade B	Grade C	Grade D	Time of follow up (months)
Zhang et al. [58]	China	2023	Side overlap	28	0	60.7% (17/28)	28	42.9% (12/28)	32.1% (9/28)	7.1% (2/28)	3.6% (1/28)	0	12
Yamashita et al. [30]	Japan	2017	Side overlap	14	0	7.1% (1/14)	10	10% (1/10)	0	10% (1/10)	0	0	6–12
Wu et al. [29]	China	2024	Modified side overlap	20	5% (1/20)	N/A	20	10% (2/20)	10% (2/20)	0	0	0	12
Morino et al. [59]	Japan	2024	Modified side overlap	17	29.4% (5/17)	N/A	17	23.5% (4/17)	N/A	N/A	N/A	N/A	12
Yamashita et al. [60]	Japan	2022	Modified side overlap	36	2.8% (1/36)	2.8% (1/36)	28	17.9% (5/28)	7.1% (2/28)	7.1% (2/28)	3.6% (1/28)	0	3–6

#### 3.1.4 Double‐Flap Technique

The double‐flap technique (DFT), also known as the Kamikawa procedure, has been reported as an improved EG after PG for the prevention of postoperative esophageal reflux by Japanese scholar Kamikawa in 1998. In DFT, the esophagus is implanted between the submucosal and muscular layers of the stomach through hand‐sewn suturing, while the anastomosis is wrapped with the hinged flaps, forming a one‐way valve (Figure 2D). Kuroda has illustrated the feasibility and safety for DFT in total complete laparoscopy through standardized surgical procedures and laparoscopic suture and ligation techniques [62, 63].

Through the addition of valvuloplasty, DFT shows a satisfactory impact on functional regurgitation, which preserves the backflow prevention of the esophago‐gastric junction (Table 4). As noted, the incidence of reflux was only 4.2% [71]. Erica and his colleagues also found a significantly lower incidence of esophagitis with DFT (3%) than EG (24%) [77]. In quantitative evaluation (reflux measured by impedance‐pH monitoring), DFT has been found to have no significant differences from preoperative values in average LES pressure and total acid exposure time [78]. Also, endoscopic examination revealed that 8.3% of patients in the DFT group suffered LA grade B or more severe reflux esophagitis vs. 13.7% in the EG group [35]. A series of studies, including multicenter studies, also found similar efficacy for DFT to prevent gastroesophageal reflux [70, 73, 74]. DFT has also been reported to attain satisfactory postoperative nutritional status and preservation of gastric remnant function [79]. In studies focused on weight loss, which was a nonnegligible consequence for patients who underwent PG, Japanese scholars found that significantly less weight was lost in patients who underwent DFT than in those who had jejunal interposition [67]. Another study also found that DFT exhibited favorable postoperative nutritional outcomes as measured with weight loss and serum albumin [68].

**TABLE 4:** The incidence of anastomotic disorders among double flap technique.

Authors	Country	Published year	Anastomotic method	Number of patients	Incidence of stenosis	Reflux symptom	Number of endoscopy	Total reflux esophagitis	Grade A	Grade B	Grade C	Grade D	Time of follow up (months)
Kuroda et al. [64]	Japan	2024	Double flap technique	38	5.3% (2/38)	N/A	38	13.2% (5/38)	7.9% (3/38)	0	2.7% (1/38)	2.7% (1/38)	12
Matsuo et al. [65]	Japan	2023	Double flap technique	80	26.3% (21/80)	12.5% (10/80)	80	10% (8/80)	N/A	10% (8/80)			12
Yu et al. [66]	Korea	2022	Double flap technique	18	11.1% (2/18)	0	18	0	0	0	0	0	12
Kumamoto et al. [67]	Japan	2021	Double flap technique	11	0	9.1% (1/11)	11	9.1% (1/11)	0	9.1% (1/11)	0	0	12
Saze et al. [68]	Japan	2021	Double flap technique	36	8.3% (3/36)	N/A	36	0	0	0	0	0	12
Kano et al. [69]	Japan	2020	Double flap technique	51	7.8% (4/51)	N/A	51	2% (1/51)	N/A	2% (1/51)			12
Tsumura et al. [70]	Japan	2020	Double flap technique	19	5.3% (1/19)	N/A	18	0	0	0	0	0	12
Hosoda et al. [35]	Japan	2019	Double flap technique	40	17.5% (7/40)	17.5% (7/40)	36	8.3% (3/36)	N/A	8.3% (3/36)			12
Shoji et al. [71]	Japan	2019	Double flap technique	147	8.3% (12/144)	N/A	144	4.2% (6/144)	0	2.1% (3/144)	2.1% (3/144)	0	12
Saeki et al. [72]	Japan	2018	Double flap technique	13	0	0	13	7.7% (1/13)	7.7% (1/13)	0	0	0	12
Kuroda et al. [73]	Japan	2018	Double flap technique	546	5.5% (30/546)	N/A	464	10.6% (49/464)	4.6% (21/464)	4.3% (20/464)	1.3% (6/464)	0.4% (2/464)	12
Hayami et al. [74]	Japan	2017	Double flap technique	43	4.7% (2/43)	N/A	43	2.3% (1/43)	0	2.3% (1/43)			12
Shibasaki et al. [75]	Japan	2017	Double flap technique	12	25% (3/12)	N/A	12	16.7% (2/12)	8.3% (1/12)	8.3% (1/12)	0	0	6
Kuroda et al. [63]	Japan	2016	Double flap technique	33	9.1% (3/33)	N/A	33	0	N/A	0	0	0	12
Li et al. [45]	China	2024	Arch‐bridge anastomosis	25	4% (1/25)	0	13	7.7% (1/13)	7.7% (1/13)	0	0	0	12
Peng et al. [76]	China	2023	Right‐sided overlap and single‐flap valvuloplasty	20	0	0^a^	20	5% (1/20)	5% (1/20)	0	0	0	12

^a^ Reflux symptom classified according to the Visick grade. Values are Visick III or more.

However, due to complicated surgical technique, the anastomotic stricture rate of DFT was relatively high. One study reported that the diameter of the esophagus less than 18 mm may be an independent risk factor for stenosis [71]. Besides, DFT requires hand‐sewn suturing, which may lead to excessive flap closure, even to postoperative stenosis. Another study recommended continuous suturing rather than interrupted suturing because of a significantly lower incidence of anastomotic stenosis (8% vs. 33%) [35]. In addition, Japanese researchers have developed stapler‐assisted DFT, which may simplify the complicated suturing procedure [80].

Another factor in the occurrence of anastomotic strictures with DFT may be the kind of operation: that is, anastomotic stricture occurred in 15% of patients who had total laparoscopic operation compared with 5% among those who had direct‐vision reconstruction through a small incision in the epigastric region [63]. Novel laparoscopic instruments such as intra‐abdominal organ retractor have been adopted to improve laparoscopic operation [81]. In a recent research, Omori and colleagues devised a modified DFT named tri double‐flap hybrid method [82]. This procedure comprised triangular linear‐stapled esophagogastrostomy and hand‐sutured flap closure, which was time‐consuming for anastomosis but achieved ideal outcomes without stenosis for all 31 patients. In order to simplify surgical procedures and maintain anti‐reflux efficacy, Li's group invented a modified valvuloplastic procedure named the arch‐bridge anastomosis. This procedure created a single seromuscular flap on the anterior wall of the stomach, and the gastric mucosal and seromuscular layers were sewed to the abdominal esophagus [45]. During one‐year postoperative follow‐up, none of the 25 patients operated with this procedure complained of reflux symptom, and among 13 patients who received endoscopic examination, only one patient had mild reflux esophagitis (LA Grade A). However, DFT is a technically demanding laparoscopic procedure due to limitation of movement and degree of freedom. Therefore, robot‐assisted PG with DFT has been used to provide a safe and convenient environment for valvuloplasty. However, the long‐term outcome with DFT needs confirmation [75]. Recently, high standard of technical requirement has still prevented DFT from coming into wide use. In addition, the risk of developing metachronous remnant gastric cancer after PG with DFT needs attention because of a reported incidence of 8.9% [83].

### 3.2 Jejunal Interposition

Jejunal interposition (JI) was first described by a Japanese surgeon in 1946 [84]. Typical JI interposes a segment of jejunum between the esophagus and the remnant stomach (Figure 2E). Based on the tolerance of digestive juice, the interposed jejunum formed a buffer zone, which avoids the gastric contents directly refluxing into the esophagus.

Typically, the longer the interposed jejunum the better is the anti‐reflux effect, but an overly long jejunum may increase the risk of food deposition and increase the difficulty on assessment of the remnant stomach [85]. Moreover, Ohyama et al. found that when gastric stump cancer was detected in patients with long interposed jejunum, the cancer likely was in an advanced stage [86]. The recommended length of jejunum is about 10 cm, which is conducive to a good anti‐reflux effect and is short enough to facilitate postoperative endoscopic evaluation [37]. Katai et al. have stated that JI is suitable and safe after PG for treatment of early gastric cancer in the upper third of the stomach [87], and Uyama et al. have firstly announced that the operation could be done feasibly and safely under laparoscopy [88]. Takayama et al. have noted that laparoscopic PG with JI has the advantages of minimal invasiveness and quicker symptomatic recovery, including shorter time to first eating and minimal postoperative inflammatory reaction as reflected by white blood cell counts and body temperature [89].

Table 5 presents evidence that JI is associated with little symptomatic esophageal reflux. Among 90 patients who received JI, none complained of reflux symptoms postoperatively, and esophagitis was found in only 1 patient during endoscopic follow‐up [94]. Katai et al. reported that only 2 of the 37 patients (5.4%) had reflux symptoms, and those two had no endoscopic esophagitis [87]. Besides, Chinese scholars have found that 0 of 31 patients with JI had reflux symptoms at 6 months after surgery, and only two patients (6.5%) had reflux esophagitis seen by endoscopic examination [99].

**TABLE 5:** The incidence of anastomotic disorders among jejunal interposition.

Authors	Country	Published year	Anastomotic method	Length of interposition jejunum (cm)	Number of patients	Incidence of stenosis	Reflux symptom	Number of endoscopy	Total reflux esophagitis	Grade A	Grade B	Grade C	Grade D	Time of follow up (months)
Gong et al. [28]	China	2023	Jejunal interposition	15	39	10.3% (4/39)	5.1%^a^ (2/39)	39	7.7% (3/39)	N/A	N/A	N/A	N/A	12
Kumamoto et al. [67]	Japan	2021	Jejunal interposition	10–13	20	0	23.5% (4/17)	17	0	0	0	0	0	12
Saze et al. [68]	Japan	2021	Jejunal interposition	N/A	10	10% (1/10)	N/A	10	10% (1/10)	N/A	N/A	N/A	N/A	12
Nomura et al. [90]	Japan	2019	Jejunal interposition	15	15	13.3% (2/15)	N/A	15	6.7% (1/15)	N/A	N/A	N/A	N/A	12
Takayama et al. [89]	Japan	2018	Jejunal interposition	15	70	1.4% (1/70)	N/A	47	2.1% (1/47)	0	0	2.1% (1/47)	0	12
Toyomasu et al. [50]	Japan	2017	Jejunal interposition	10–15	40	12.5% (5/40)	N/A	40	10% (4/40)	5% (2/40)	5% (2/40)	0	0	12
Tao et al. [91]	China	2016	Jejunal interposition	15–25	15	0	6.7%^a^ (1/15)	15	100% (15/15)	80% (12/15)	13.3% (2/15)	6.7% (1/15)	0	12
Yasuda et al. [51]	Japan	2015	Jejunal interposition	15	21	10% (2/20)	5% (1/20)	17	23.5% (4/17)	23.5% (4/17)	0	0	0	12
Ohashi et al. [92]	Japan	2015	Jejunal interposition	10	65	9.2% (6/65)	35.4% (23/65)	65	0	0	0	0	0	12
Nomura et al. [93]	Japan	2014	Jejunal interposition	15	10	20% (2/10)	0	10	10% (1/10)	N/A	N/A	N/A	N/A	12
Nakamura et al. [36]	Japan	2014	Jejunal interposition	10–15	25	31.8% (7/22)	N/A	22	4.5% (1/22)	4.5% (1/22)	0	0	0	12
Kinoshita et al. [94]	Japan	2013	Jejunal interposition	15	90	6.7% (6/90)	0	81	1.2% (1/81)	N/A	N/A	N/A	N/A	6
Nozaki et al. [85]	Japan	2013	Jejunal interposition	N/A	102	5.9% (6/102)	N/A	95	3.2% (3/95)	1.1% (1/95)	1.1% (1/95)	0	1.1% (1/95)	12
Takagawa et al. [95]	Japan	2010	Jejunal interposition	10	19	21.1% (4/19)	5.3% (1/19)	19	15.8% (3/19)	N/A	N/A	N/A	N/A	12
Katai et al. [96]	Japan	2010	Jejunal interposition	10–23	128	10.2% (13/128)	5.5% (7/128)	118	1.7% (2/118)	N/A	N/A	N/A	N/A	N/A
Shinohara et al. [97]	Japan	2006	Jejunal interposition	N/A	18	0	50% (9/18)	18	77.8% (14/18)	72.2% (13/18)	0	5.6% (1/18)	0	12
Hoshikawa et al. [98]	Japan	2001	Jejunal pouch	N/A	18	0	27.8% (5/18)	18	0	0	0	0	0	12
Adachi et al. [53]	Japan	1999	Jejunal interposition	N/A	16	6.3% (1/16)	0	16	0	0	0	0	0	N/A

^a^ Reflux symptom classified according to the Visick grade. Values are Visick III or more.

Thus, it appears that the jejunal loop largely prevents reflux of gastric content into the esophagus. Nonetheless, JI has problems that limit its wide application: the procedure is time‐consuming and technically complicated, with at least three anastomoses and accompanying risk of postoperative anastomotic stenosis, delayed gastric emptying [88]. Besides, in several studies, interposed jejunum may cause difficulty in endoscopic surveillance, particularly in cases with tortuosity or overlength of the interposed segment [37].

### 3.3 Double‐Tract Reconstruction

Double‐tract reconstruction (DTR), a type of continuous jejunal interposition, was first reported by Japanese surgeon Aikou and associates in 1988 [100]. It involves placing a segment of the jejunum between the esophagus and residual stomach, maintaining the gastroduodenal passage while creating a continuous jejunal channel (Figure 2F). This procedure has improved gastric emptying and decreased the incidence of gastric retention [101]. In addition, DTR facilitates the passage of food through the duodenum, stimulates the release of cholecystokinin and secretin, and enhances the digestion and absorption of nutrients.

In recent years, many scholars have investigated the clinical effects of DTR (Table 6). The Korean surgeon Ahn and his team reported that under laparoscopic PG with DTR reconstruction, only 2 of 43 patients (4.6%) had mild reflux symptoms (Visick grade II); 2 cases of anastomotic stenosis were resolved with balloon dilatations; and postoperative nutritional status (according to body weight and serum albumin values) was satisfactory [113]. Another Korean team reported that 0–16 patients with DTR had reflux symptoms at an average of 9‐month follow‐up [115]. A retrospective study on PG with DTR of 92 patients from China found that 6 patients (6.5%) had LA grade A reflux esophagitis; 2 (2.2%) had LA grade B at 1 year postoperatively; and none of these patients had anastomotic stenosis [107]. In a Japanese study, the incidence of reflux symptoms after one‐year follow‐up, among patients in the DTR group was 10.5%, significantly lower than EG group (54.5%), with no anastomotic stricture identified. Two patients had esophagitis diagnosed endoscopically (1 LA grade B and 1 grade C), and postoperative nutritional status showed no significant difference between the DTR and the EG group [31].

**TABLE 6:** The incidence of anastomotic disorders among double tract reconstruction.

Authors	Country	Published year	Anastomotic method	Length of interposition jejunum (cm)	Number of patients	Incidence of stenosis	Reflux symptom	Number of endoscopy	Total reflux esophagitis	Grade A	Grade B	Grade C	Grade D	Time of follow up (months)
Xu et al. [102]	China	2024	Double tract	10–15	50	6% (3/50)	16%^a^ (8/50)	50	12% (6/50)	N/A	N/A	N/A	N/A	12
Hasegawa et al. [103]	Japan	2024	Double tract	N/A	39	0	0	39	0	0	0	0	0	12
Wang et al. [25]	China	2024	Double tract	10–15	87	1.1% (1/87)	9.2% (8/87)	87	9.2% (8/87)	N/A	N/A	N/A	N/A	N/A
Wu et al. [61]	China	2024	Double tract	10–15	23	5% (1/20)	N/A	23	39.1% (9/23)	21.7% (5/23)	17.4% (4/23)	0	0	12
Morino et al. [59]	Japan	2024	Double tract	15–20	11	9.1% (1/11)	N/A	11	9.1% (1/11)	N/A	N/A	N/A	N/A	12
Li et al. [104]	China	2023	Double tract	12–15	50	0	0^a^	50	4% (2/50)	4% (2/50)	0	0	0	12
Zhang et al. [105]	China	2023	Double tract	15	36	0	N/A	36	8.3% (3/36)	8.3% (3/36)	0	0	0	12
Xu et al. [46]	China	2023	Double tract	10–15	55	5.5% (3/55)	14.5%^a^ (8/55)	55	10.9% (6/55)	N/A	N/A	N/A	N/A	12
Chen et al. [27]	China	2023	Double tract	15	36	5.6% (2/36)	19.4% (7/36)	36	13.9% (5/36)	5.6% (2/36)	5.6% (2/36)	2.8% (1/36)	0	12
Ma et al. [106]	China	2022	Double tract	15–20	33	6.1% (2/33)	6.5%^a^ (2/31)	29	100% (29/29)	93.1% (27/29)	6.9% (2/29)	0	0	12
Hosogi et al. [47]	Japan	2022	Double tract	N/A	12	0	N/A	9	22.2% (2/9)	0	11.1% (1/9)	11.1% (1/9)	0	12
Yu et al. [66]	Korea	2022	Double tract	15	51	2% (1/51)	N/A	51	5.9% (3/51)	2% (1/51)	3.9% (2/51)	0	0	12
Ma et al. [107]	China	2021	Double tract	12–15	100	1% (1/100)	0^a^	92	8.7% (8/92)	6.5% (6/92)	2.2% (2/92)	0	0	12
Zhang et al. [108]	China	2021	Double tract	12–15	113	1% (1/113)	0^a^	105	5.7% (6/105)	4.8% (5/105)	1% (1/105)	0	0	12
Xiao et al. [109]	China	2021	Double tract	10–15	110	1% (1/110)	N/A	80	11.3% (9/80)	8.8% (7/80)	2.5% (2/80)	0	0	12
Sato et al. [110]	Japan	2021	Double tract	10–12	75	0	0	75	8% (6/75)	N/A	8% (6/75)			36
Eom et al. [29]	Korea	2021	Double tract	15	58	8.6% (5/58)	N/A	58	3.4% (2/58)	1.7% (1/58)	1.7% (1/58)		N/A	12
Tominaga et al. [34]	Japan	2021	Double tract	15	17	0	5.9% (1/17)	17	5.9% (1/17)	N/A	5.9% (1/17)			12
Saze et al. [68]	Japan	2021	Double tract	N/A	14	21.4% (3/14)	N/A	14	21.4% (3/14)	N/A	N/A	N/A	12	12
Choi et al. [111]	Korea	2020	Double tract	10–15	37	0	N/A	33	0	0	0	0	0	N/A
Ko et al. [112]	Korea	2020	Double tract	12–15	52	2% (1/52)	0^a^	52	3.8% (2/52)	3.8% (2/52)	0	0	0	12
Nomura et al. [90]	Japan	2019	Double tract	15	15	13.3% (2/15)	N/A	15	6.7% (1/15)	N/A	N/A	N/A	N/A	12
Aburatani et al. [31]	Japan	2017	Double tract	15	19	0	10.5% (2/19)	19	10.5% (2/19)	0	5.3% (1/19)	5.3% (1/19)	0	12
Ahn et al. [113]	Korea	2014	Double tract	10	43	4.7% (2/43)	4.7% (2/43)	43	0	0	0	0	0	3
Nomura et al. [93]	Japan	2014	Double tract	15	10	10% (1/10)	10% (1/10)	10	10% (1/10)	N/A	N/A	N/A	N/A	12
Li et al. [104]	China	2023	Piggyback jejunal interposition double‐tract reconstruction	12–15	50	0	0^a^	50	4% (2/50)	4% (2/50)	0	0	0	12
Hong et al. [114]	China	2016	Modified double tract	15–20	21	0	4.8% (1/21)	17	0	0	0	0	0	6

^a^ Reflux symptom classified according to the Visick grade. Values are Visick III or more.

The interposed jejunum may serve as a buffer mechanism, slowing the rate of food ingestion and resistance to both acidic gastric and alkaline digestive juices. This resistance might mitigate the stimulation of gastric acid secretion at the anastomotic site, thereby reducing the likelihood of anastomotic stenosis and leakage. Simultaneously, jejunal interposition exhibits minimal constraints on the size of the residual stomach and is applicable to most reconstructions after PG. Theoretically, a longer interstitial jejunal loop corresponds to a more effective anti‐reflux outcome; however, excessively long loops (30–40 cm) may impede post‐surgery endoscopic procedures and increase the likelihood of food retention in the intestine. Consequently, it has been suggested that the ideal length for interstitial jejunal loop is 10–15 cm [116]. Despite the demonstrated efficacy of DTR in preventing reflux, this procedure presents challenges, such as the need for multiple anastomoses, technical complexity, prolonged surgical time, and increased costs. Furthermore, around 40% of ingested food may bypass the gastroduodenal pathway in the gastrointestinal tract at 2 years postoperatively, potentially resulting in malnutrition [117]. However, if only a minority of food traverses the gastroduodenal pathway, postoperative functional outcomes may not be significantly different from TG. The underutilization of the gastric pathway could impede the widespread acceptance and implementation of this surgical technique [93]. In addition, recent study indicated that DTR after PG is a risk factor for postoperative hypoglycemia, especially nocturnal hypoglycemia, in non‐diabetic GC patients, which may hinder the further development [118].

## 4 Discussion

The evidence base for reconstruction following PG consists primarily of single‐center retrospective studies, along with a limited number of multi‐center trials. Overall, the quality of this evidence ranges from moderate to low and is associated with inherent methodological limitations. Many studies are characterized by small sample sizes—often including only a few dozen patients per reconstruction method—and short follow‐up periods, which restrict statistical power and limit the ability to detect differences in long‐term outcomes. Selection bias represents a significant concern: in non‐randomized studies, the choice of reconstruction technique is frequently influenced by surgeon preference or patient‐specific factors. For instance, surgeons may opt for technically simpler anastomoses in older or higher‐risk patients, while reserving more complex approaches, such as the double‐flap method, for younger, lower‐risk individuals. Such imbalances may confound outcome comparisons across techniques. Furthermore, there is considerable heterogeneity in outcome reporting. While some studies prioritize objective endpoints—such as endoscopic incidence of reflux esophagitis or stricture requiring dilation—others emphasize subjective symptom scores or nutritional parameters, thereby complicating direct comparisons across studies. One of the primary goals of adopting proximal gastrectomy over total gastrectomy is to improve patient‐centered outcomes, such as postoperative nutritional status, symptom burden, and overall quality of life (QOL). Our review found that reconstruction techniques can significantly influence these outcomes, although results across studies have been mixed. Importantly, formal QOL instruments tailored to post‐gastrectomy patients (like PGSAS‐45) have not been widely applied in head‐to‐head comparisons of reconstruction methods. The majority of studies rely on general or cancer‐specific QOL surveys or report only symptom frequencies. The PGSAS‐45, which comprehensively assesses multiple domains of post‐gastrectomy life (including meal‐related distress, functional symptoms, mental health, and social functioning), could provide deeper insights. Applying such detailed QOL assessments to compare, say, DTR vs. double‐flap vs. jejunal interposition, would likely elucidate nuanced differences and trade‐offs between these techniques that simpler metrics might miss. We encourage future studies to incorporate standardized patient‐reported outcome measures like PGSAS‐45 to capture the full impact of each reconstruction on patients' lives.

The aim of digestive tract reconstruction after PG is to preserve residual gastric function to the maximum extent possible, rebuild the anatomical structures of the gastroesophageal junction and maintain the normal physiological passage of food through the duodenum on the basis of radical treatment of tumors. If these goals are reached, the incidence of anastomosis‐related complications may decrease, and postoperative nutritional status and quality of life of patients likely can be maintained. At the same time, the procedure should be as simple and safe as possible and permit convenient postoperative endoscopic examination. Our review makes it clear that no single reconstruction method is ideal for every patient. Each of the prevalent techniques has its role, with specific strengths and limitations:

EG, which is considered the simplest method, should ideally be combined with anti‐reflux measures if used. The reflux rate without modified EG is unacceptable. Adding DFT or SOFY to EG can form an effective anti‐reflux barrier, reducing the postoperative reflux rate to about 10% or lower. Traditional valveless EG should only be considered when other options are not feasible.

JI, which is conducted by inserting a segment of jejunum between the esophagus and the stomach, provides a reasonable anti‐reflux mechanism and maintains the continuity of the duodenum. JI is applicable to most PG cases, does not limit the size of the gastric stump, and is beneficial for nutrition. However, it requires two anastomoses, which can prolong the operation time. Esophageal jejunostomy stenosis is rare, and the reflux rate is usually low, making it a reliable choice. Clinically, JI is recommended when the gastric stump is large enough, has good mobility, and the tension of the esophagogastric anastomosis is high.

DTR, considered to be one of the most popular techniques currently, effectively combines JI with Roux‐en‐Y. By sending part of the food through the jejunal loop to the duodenum while performing an esophagus‐to‐gastric stump anastomosis, DTR achieves excellent reflux control and ensures a certain physiological duodenal pathway. Our review found that DTR is the most frequently reported reconstruction in recent literature, consistently showing a low reflux rate and acceptable nutrition. DTR involves multiple anastomoses and is slightly more complex than a single EG, but many laparoscopic surgeons adopt it due to its repeatability. We recommend DTR as the first‐line reconstruction after PG, especially for minimally invasive surgeries, as it balances technical feasibility and functional benefits.

## 5 Conclusion

In conclusion, PG provides clear quality‐of‐life benefits for patients with early upper gastric cancer, provided that meticulous reconstruction is carried out. Surgeons now have a variety of techniques at their disposal, which can be customized according to the patient's needs. Although the “best” reconstruction method remains a subject of debate, it is clear that methods that preserve the duodenal passage and prevent reflux (such as DTR and DFT) achieve the most favorable outcome balance. Ongoing research and innovation are expected to further improve these techniques and may discover new solutions to address long‐term challenges [119, 120, 121, 122]. From a broader perspective, the optimal reconstruction after PG remains an open question. Our review emphasizes the necessity of further research. There are still several gaps and opportunities for future studies: Before definitive data are available, our clinical recommendation is that surgeons should focus on mastering one or two preferred reconstruction methods that are safe to perform, always including anti‐reflux modifications, and closely follow up on patients' nutritional and functional outcomes. Through iterative improvements and sharing of scientific evidence, we are approaching the ultimate goal: not only enabling survival of patients with gastric cancer but also allowing them to enjoy a strong and healthy life after surgery.

## Author Contributions

**Linchuan Li:** data curation (equal), formal analysis (equal), formal analysis (equal), funding acquisition (lead), funding acquisition (lead), visualization (lead), visualization (lead), writing – original draft (lead), writing – original draft (lead). **Dexu Zhang:** data curation (equal), investigation (equal), visualization (equal), writing – original draft (equal). **Siyi Song:** data curation (equal), investigation (equal), writing – original draft (equal). **Shuohui Dong:** data curation (equal), resources (equal), writing – review and editing (equal). **Qian Xu:** resources (equal), validation (equal), writing – review and editing (equal). **Guangyong Zhang:** conceptualization (equal), supervision (equal), visualization (equal). **Jiankang Zhu:** conceptualization (equal), supervision (equal), validation (equal), visualization (equal).

## Funding

This work was supported by the Natural Science Foundation of Shandong Province (Grant ZR2022QH322).

## Conflicts of Interest

The authors declare no conflicts of interest.

## Data Availability

The data that support the findings of this study are available from the corresponding author upon reasonable request.
